# Euchromatin histone methyltransferase 1 regulates cortical neuronal network development

**DOI:** 10.1038/srep35756

**Published:** 2016-10-21

**Authors:** Marijn Bart Martens, Monica Frega, Jessica Classen, Lisa Epping, Elske Bijvank, Marco Benevento, Hans van Bokhoven, Paul Tiesinga, Dirk Schubert, Nael Nadif Kasri

**Affiliations:** 1 Department of Neuroinformatics, Radboud University Nijmegen, Faculty of Science, Heyendaalseweg 135, 6525 AJ Nijmegen, the Netherlands; 2 Donders Institute for Brain, Cognition and Behaviour, P.O. Box 9101, 6500 HB, Nijmegen, the Netherlands; 3 Department of Cognitive Neuroscience, Radboudumc, P.O. Box 9101, 6500 HB, Nijmegen, the Netherlands; 4 Department of Human Genetics, Radboudumc, P.O. Box 9101, 6500 HB, Nijmegen, the Netherlands

## Abstract

Heterozygous mutations or deletions in the human Euchromatin histone methyltransferase 1 (*EHMT1*) gene cause Kleefstra syndrome, a neurodevelopmental disorder that is characterized by autistic-like features and severe intellectual disability (ID). Neurodevelopmental disorders including ID and autism may be related to deficits in activity-dependent wiring of brain circuits during development. Although Kleefstra syndrome has been associated with dendritic and synaptic defects in mice and *Drosophila*, little is known about the role of EHMT1 in the development of cortical neuronal networks. Here we used micro-electrode arrays and whole-cell patch-clamp recordings to investigate the impact of EHMT1 deficiency at the network and single cell level. We show that EHMT1 deficiency impaired neural network activity during the transition from uncorrelated background action potential firing to synchronized network bursting. Spontaneous bursting and excitatory synaptic currents were transiently reduced, whereas miniature excitatory postsynaptic currents were not affected. Finally, we show that loss of function of EHMT1 ultimately resulted in less regular network bursting patterns later in development. These data suggest that the developmental impairments observed in EHMT1-deficient networks may result in a temporal misalignment between activity-dependent developmental processes thereby contributing to the pathophysiology of Kleefstra syndrome.

Intellectual disability (ID) affects 2–3% of the population and is characterized by an intelligence quotient (IQ) below 70 and an impairment of normal adaptive behavior that is evident before the age of 18^1^,^2^. ID disorders are phenotypically heterogeneous and have been associated with a large number of genes^3^. ID has since long been proposed to be “a disease of the synapse”, with the underlying assumption that synaptic malfunction can severely affect network connectivity^4^,^5^,^6^,^7^. This is supported by the observation that genes associated with ID converge on common signaling cascades that impinge on synaptic function. Examples include pathways involved in synaptic plasticity, Rho- and Ras-GTPase signalling and, more recently, epigenetic regulation^8^,^9^. In accordance with the correlation between epigenetic dysregulation and ID, evidence is accumulating that epigenetic actions regulate synaptic function and memory^10^,^11^,^12^.

Kleefstra syndrome (KS) is a neurodevelopmental disorder caused by the haploinsufficiency of the human Euchromatin histone methyltransferase 1 (*EHMT1*) gene^13^,^14^. KS is characterized by ID, general developmental delay, childhood hypotonia, craniofacial abnormalities and autistic-like behavioral problems. In addition, mutations in *EHMT1* have also been associated with isolated idiopathic autism spectrum disorder (ASD)^15^ and schizophrenia^16^. EHMT1 (also known as GLP) together with its paralog EHMT2 (also known as G9a) and other proteins is found in chromatin remodeling complexes that catalyze the dimethylation of histone H3 at lysine 9 (H3K9me2), a post-translational modification associated with repression of gene transcription^17^. H3K9me2 is an epigenetic mark that is dynamically regulated and has been associated with cognition in mouse and *Drosophila*^18^,^19^,^20^,^21^,^22^. Consequently, genetic and pharmacological manipulations of EHMT1 and EHMT2 *in vivo,* in mice and *Drosophila,* have shown deficits in learning and memory^18^,^19^,^20^,^21^,^22^.

At the cellular level, hippocampal CA1 neurons of *Ehmt1*^+/−^ mice showed reduced dendritic branching and spine density^19^. Similar neuronal phenotypes were also observed in *Drosophila* lacking *EHMT*, which could be rescued by reintroduction of EHMT in adult flies^21^. In addition, *Ehmt1*^+/−^ mice showed increased paired-pulse facilitation at the CA3-CA1 synapse, indicative for a presynaptic deficit associated with reduced release probability^19^. This study further revealed that miniature excitatory postsynaptic currents (mEPSC) frequencies, but not their amplitudes, were significantly reduced in *Ehmt1*^+/−^ CA1 pyramidal neurons. More recently, EHMT1 was shown to be critical for the repression of BDNF during synaptic scaling up^23^. As a consequence, loss of EHMT1 prevented synaptic scaling up *in vitro* and *in vivo*. Although these results point toward a synaptic deficit, it is still unknown what the impact is of EHMT1 deficiency during critical periods of early postnatal development at the level of cortical network activity. Increasing evidence suggests that there are sensitive time periods during development in which a neuronal network is susceptible for disturbances caused by the deficiency of genes or environmental factors^24^,^25^,^26^. Developmental impairments could result in a temporal misalignment between different activity-dependent developmental processes leading to neuronal network miswiring.

Here, we used micro-electrode arrays (MEAs) and whole-cell patch-clamp recordings to measure network and single cell activity during neuronal network development in cortical cultures. Cultured neurons grown on MEAs typically self-assemble into functionally connected networks that exhibit isolated spontaneous action potentials as well as periodical synchronized bursts of action potentials^27^. Recently, monitoring the properties of spontaneous network activity of self-organizing *in vitro* neuronal networks over time was shown to be a valuable tool for investigating the role of specific genes in neuronal network formation^28^,^29^,^30^,^31^,^32^. However, until now, few studies have measured the impact of loss of ID-associated genes during neuronal development^33^,^34^. We utilized RNA interference to reduce EHMT1 expression in cortical neurons grown in culture. Using MEA and patch-clamp recordings, we found that EHMT1 deficiency impaired spontaneous network activity and lowered firing rates during early development, whereas basal, action potential-independent excitatory synaptic transmission was unaffected. The development to a network state of synchronized bursting activity in EHMT1-deficient neuronal networks was delayed by several days compared to control networks. Furthermore, we found that later in development EHMT1 deficiency led to network activity with increased irregularity in the timing of network bursts. These data indicate that EHMT1 is required for proper cortical neural circuitry development.

## Results

### EHMT1 is required for neural network activity during development

**Figure 1:**
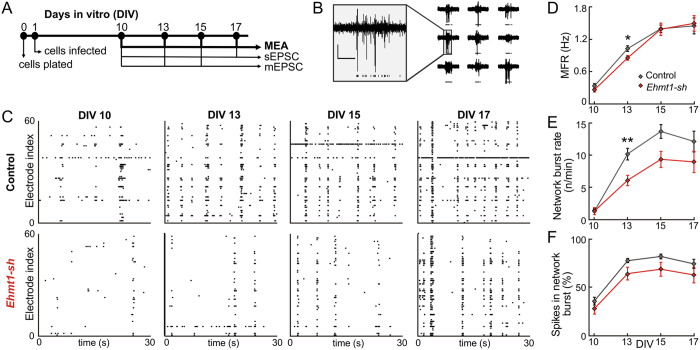
Neural network activity during development. (**A**) Schematic representation of the time course of experiments. (**B**) Example MEA traces of extracellular action potentials on 9 adjacent electrodes. Detected action potentials are denoted with a black tick below each trace. Scale bar is 20 μV (vertical) and 100 ms (horizontal) (**C**) Raster plots of action potential activity across 60 MEA electrodes. Each detected action potential is indicated as a black tick. (**D**) The mean firing rate (MFR) is the average action potential frequency that is detected on the active electrodes, plotted at different DIVs. (**E**) The burst rate, defined as synchronous network activity for which the spike density crossed a predefined threshold, was plotted for different DIVs. (**F**) Amount of action potentials that fall within bursts as a percentage of the total number of detected action potentials are plotted at different DIVs. Data are means ± SEM. Statistics were based on n = 24 (DIV 10), 25 (DIV 13), 25 (DIV 15) and 24 (DIV 17) recordings for control and n = 19 (DIV 10), 19 (DIV 13), 19 (DIV 15) and 18 (DIV 17) for Ehmt1-sh DIV; *denotes p < 0.05, **denotes p < 0.01 (Mann–Whitney test, with p-values corrected for multiple comparisons using the false discovery rate method, see Supplemental Table S1).

We initially investigated how EHMT1 deficiency in neurons affected the development of neuronal population activity. To this end we monitored spontaneous activity in dissociated cortical wild-type (WT, control) cultures on MEAs and in cultures in which EHMT1 expression was down regulated by approximately 55% (Fig. S1) through RNA interference during development. We infected cortical neurons with a previously validated lentivirus expressing a short hairpin RNA (shRNA) against EHMT1 (*Ehmt1-sh*)^23^ at 1 day *in vitro* (DIV) and recorded extracellular activity in individual cultures over time at DIV 10, 13, 15 and 17 (Fig. 1A) from 60 electrodes in the MEAs (Fig. 1B). The pattern of spontaneous activity of the neuronal networks consisted of background (spatially and temporally isolated) action potentials and network bursts (i.e. local and global synchronized bursts that can be detected in most of the recording channels) (Fig. 1C). Within 10 days of plating, spontaneous activity could be reliably recorded (Fig. 1C) in control cultures on MEAs. As development progressed, we found that the firing rate and frequency of network bursting increased, reaching stationary levels at DIV 15. Neuronal network activity at DIV 10 has been reported to be mostly spatially and temporally uncorrelated, and a transition to the typical state of repetitive synchronized firing occurs after two weeks *in vitro* for normal cortical cultures^28^,^35^,^36^. We recorded a modest reduction of 18% in the mean firing rate (MFR) for the EHMT1-deficient networks compared to the control networks during the early developmental stage of DIV 13 (p = 0.037, Fig. 1D). Furthermore, the activation in the number of electrodes (MFR > 0.1 Hz) was delayed during development, with fewer active electrodes at DIV 13 (p = 0.01) and DIV 15 (p = 0.04) and no significant difference in the number of active electrodes at DIV 17 (p > 0.05). Interestingly, later in development (i.e. DIV 17) EHMT1-deficient networks reached firing rates that were comparable to the control networks, suggesting that loss of EHMT1 early in development leads to delayed network formation. In terms of burst generation we found that the rate of synchronized bursting at DIV 13 was severely reduced (40%, p = 0.003, Fig. 1E).

Next, we investigated whether during development the control and EHMT1-deficient networks changed the ratio between action potentials belonging to background activity and action potentials belonging to network bursts^27^. At DIV 10 the majority (64% for control and 71% for EHMT1-deficient networks) of action potentials were background action potentials, while at DIV 13 and later the majority (78% for control and 65% for EHMT1-deficient networks) of action potentials belonged to network bursts (Fig. 1F). This typical profile was present for both conditions, and the ratio of network bursts to background action potentials was comparable for all tested time points (Fig. 1F).

**Figure 2:**
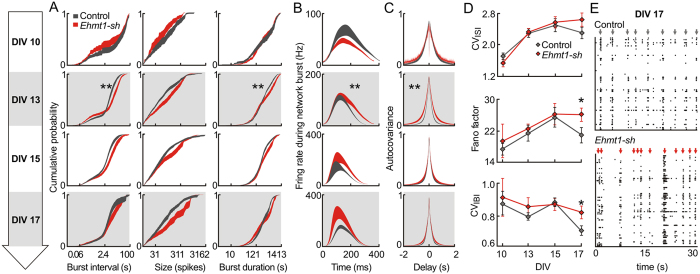
Characterization of burst dynamics. (**A**) Normalized cumulative distribution of the interburst intervals, burst size and burst duration at indicated DIVs. (**B**) To obtain the average profiles of firing rates during bursts, each detected burst was aligned to its onset. Network bursts were significantly longer at DIV 13 (statistics as in A, third panel). (**C**) The normalized autocovariance of action potentials was calculated per electrode and averaged over the active electrodes for each recording. (**D**) Measures of spike and burst irregularity: coefficient of variation for the interspike intervals (CV_ISI_), Fano Factor (FF) and the coefficient of variation for the interburst intervals (CV_IBI_). (**E**) Example raster plots showing increased burst irregularity for Ehmt1-sh at DIV17. Network bursts are marked by arrows mark. Data are means ± SEM. Statistics were based on n = 24 (DIV 10), 25 (DIV 13), 25 (DIV 15) and 24 (DIV 17) recordings for control and n = 19 (DIV 10), 19 (DIV 13), 19 (DIV 15) and 18 (DIV 17) for *Ehmt1-sh*; *denotes p < 0.05, **denotes p < 0.01 (Mann–Whitney test, with p-values corrected for multiple comparisons using the false discovery rate method, see Supplemental Table S1).

Consistent with the reduced rate of network bursts at DIV 13 in EHMT1-deficient networks, the interburst intervals (IBIs; i.e. the interval between two consecutive network bursts) were significantly longer (39%) at DIV 13 (p = 0.005, Fig. 2A). Although at DIV 13 fewer network bursts occurred for the EHMT1-deficient condition, these bursts lasted longer (24%, p = 0.001, Fig. 2A,B). The mean burst size (i.e. number of action potentials per network burst), however, was not different (Fig. 2A). Taken together, at DIV 13 the overall firing rate was reduced and fewer network bursts occurred in the EHMT1-deficient networks. Furthermore, when a network burst occurred, the propagation of activity through the network was prolonged.

To further corroborate our results that EHMT1 is required for normal network development and exclude off-targets effects that can be caused by the shRNA, we also performed experiments using cultures derived from wild-type (WT, n = 12) and *Ehmt1*^+/−^ mice (n = 10). The firing rate and frequency of bursting increased in the WT condition, reaching stationary levels at DIV 15 (see Supplemental Fig. S2A,B). Networks derived from *Ehmt1*^+/−^ mice showed a delay in the neuronal network maturation. The level of activity (i.e. firing and bursting rate) was significantly lower compared to WT at DIV 13, 15 and 17. Interestingly, the level of activity of EHMT1-deficient networks still increased during development, reaching the same level of WT condition at DIV 20. Our results show a similar phenotype using *Ehmt1* shRNAs and *Ehmt1*^+/−^ mice and thus strongly indicate that the loss of function of EHMT1 leads to a delay in neural network development. Since EHMT1 has been shown to play a critical role during embryonic development we chose to continue the analysis of neural network development using *Ehmt1* shRNAs.

### EHMT1 is required for action potential coherency during early development

The dynamics of synchronized bursting depend on the degree of functional synaptic connectivity, which is influenced by the properties of both the pre- and postsynaptic components^31^,^37^ as well as the structure of the neuronal connectivity^38^,^39^. During culture development, synaptic transmission changes from action potential-independent vesicle release to predominantly action potential-dependent release^40^. Action potential propagation is fast for networks that have vesicle release locked to the presynaptic action potential at short latency. We assessed the time-scale of the action potential covariance to quantify to what extent action potentials occurred within a short time window of other action potentials. After a few days in culture, neurons started to connect to each other through functionally active synapses. At DIV 10, both control and EHMT1-deficient neuronal networks exhibited electrophysiological activity mainly composed of background action potentials. The level of synchronization of the network activity was low in both conditions (Fig. 2C). From DIV 13 onwards, the control network exhibited patterns of synchronized activity. In contrast, EHMT1-deficient networks show a delay in the development of synchronized activity: at DIV 13 the action potential autocovariance half-width was higher as compared to control networks (40%, p = 0.001, Fig. 2C), indicating a lower degree of connectivity in the EHMT1-deficient networks The difference in autocovariance between conditions was not significant at the later developmental stages (Fig. 2C). These results imply that EHMT1 deficiency led to a delay in the action potential coherency during early network development.

### EHMT1 deficiency leads to increased irregularity in burst timing later in development

Cultured neurons develop towards a typical state of periodic, synchronized bursting^36^,^41^. To investigate whether EHMT1 deficiency had an effect on more complex network activity, we determined the irregularity of the interspike intervals (ISIs) and interburst intervals (IBIs). The coefficient of variation of the ISI (CV_ISI_) is a measure of spike irregularity. We did however not observe significant changes in the CV_ISI_, indicating no difference in irregularity in the spike timing during development (Fig. 2D). Furthermore, we measured the irregularity of spike timing by calculating the Fano Factor (FF) of the spike trains, averaged over the individual channels (Fig. 2D). We found that at DIV 17 the FF was higher for EHMT1-deficient networks compared to the control networks (20%, p = 0.04, Fig. 2D). We hypothesized that the increased FF was caused by an increased irregularity in the network burst timing. Indeed, the EHMT1-deficient networks also had more irregular IBIs, as indicated by an increased value for the coefficient of variation of the IBI (CV_IBI_), (p = 0.049, Fig. 2D), and a 20% increase in the rate-independent metric for network burst irregularity (p = 0.027)^42^. Representative raster plots showing the increased irregularity in EHMT1-deficient networks are shown in Fig. 2E. Taken together, the increase in FF and CV_IBI_ show that EHMT1 deficiency resulted in an increased network burst irregularity at DIV 17.

### EHMT1 is required for action potential-dependent inputs during early development

**Figure 3:**
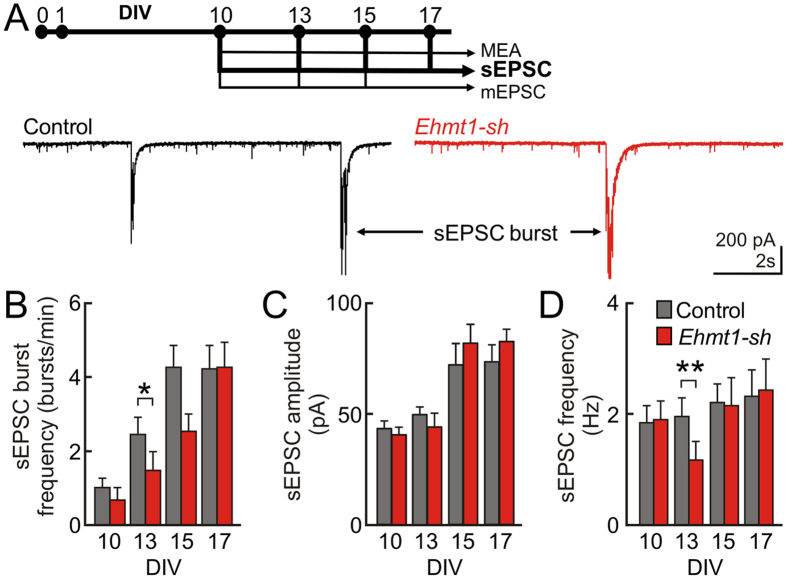
Action potential-dependent inputs during early development. (**A**) *Top*, Schematic representation of the time course of experiments. *Down*, representative example traces at DIV 13 showing sEPSCs. (**B**) sEPSC-burst frequency at different DIVs. (**C**,**D**) The amplitude (**C**) and frequency (**D**) of sEPSCs. Data are means ± SEM. Statistics were based on n = 17 (DIV 10), 20 (DIV 13), 20 (DIV 15) and 19 (DIV 17) recordings for control and n = 17 (DIV 10), 21 (DIV 13), 18 (DIV 15) and 19 (DIV 17) for *Ehmt1-sh*; *denotes p < 0.05, **denotes p < 0.01 (Mann–Whitney test, with p-values corrected for multiple comparisons using the false discovery rate method, see Supplemental Table S1).

Our results at the network level imply that EHMT1-deficient networks show a delayed onset of background and network burst activity generation. We investigated whether this delay in generating activity was reflected in the overall amount of synaptic inputs driving the individual neurons. Using whole-cell recordings we recorded intrinsic properties (DIV 13, in current clamp, Supplemental Table S2) and spontaneous excitatory postsynaptic current (sEPSC, in voltage clamp at DIV 10, 13, 15 and 17). All measured parameters were similar except for the input resistance and Rheobase, which were increased and decreased respectively in *Ehmt1-sh* neurons (Supplemental Table S2). It is however unlikely that these changes contribute to the hypo-activity observed in EHMT1-deficient neural networks since they would make the *Ehmt1-sh* neurons more excitable. At the level of sEPSCs we typically observed bursts of sEPSCs, which, due to the synchronized action potential input, resulted in input currents that saturated the amplifier (Fig. 3A). The network sEPSC-burst rate so inferred was reduced by 41% in the EHMT1-deficient networks as compared to control conditions at DIV 13 (p = 0.022, Fig. 3B), which is consistent with the MEA population recordings reported in the preceding section. The properties of sEPSCs and their base frequency were measured during the IBIs. We found that the amplitude of the synaptic inputs for EHMT1-deficient networks was not different as compared to the control networks at any of the tested time points (Fig. 3C). In contrast, the frequency of sEPSCs was reduced by 40% as compared to control conditions at DIV 13 (p = 0.004, Fig. 3D). Together these data show that, besides a significant reduction in burst occurrences, neurons in EHMT1-deficient networks receive a reduced amount of excitatory synaptic inputs at DIV 13. Again this reduction in sEPSC frequency was transient since later during network development, i.e. DIV 15 and 17, significant differences in frequency were absent.

**Figure 4:**
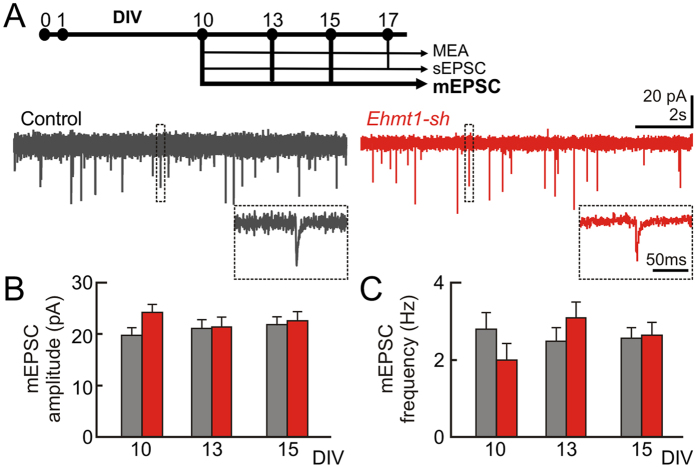
Action potential-independent inputs during early development. (**A**) Miniature excitatory postsynaptic currents (mEPSC) were recorded for DIV 10, 13 and 15 in the presence of tetrodotoxin to block action potentials and picrotoxin to block inhibitory events. Representative traces of a whole-cell voltage-clamp recordings show mEPSC events. (**B**) The mEPSC amplitude and (**C**) mEPSC frequency plotted as function of DIV. Data are means ± SEM. Statistics were based on n = 18 (DIV 10), 20 (DIV 13) and 20 (DIV 15) recordings for control and n = 17 (DIV 10), 20 (DIV 13) and 21 (DIV 15) for *Ehmt1-sh*; *denotes p < 0.05, **denotes p < 0.01 (Mann–Whitney test, with p-values corrected for multiple comparisons using the false discovery rate method, see Supplemental Table S1).

Finally, we compared the extent and efficiency of synaptic connections between cells of the EHMT1-deficient and the control networks by means of mEPSC, which depend on stochastic release of individual presynaptic vesicles and not on presynaptic action potential firing. Our previous results imply that most relevant differences in network behavior occur at around DIV 13. Therefore, for these experiments we focused on using whole-cell voltage-clamp recordings in the presence of tetrodotoxin and picrotoxin at DIV 10, 13 and 15 (Fig. 4A). Remarkably, EHMT1 deficiency did not significantly change the frequency (Fig. 4B) or the amplitude of mEPSCs (Fig. 4C) at any of the three developmental time points tested.

Taken together, our single cell data also indicate that EHMT1 deficiency led to a transient reduction in overall firing and burst rates at DIV 13, while there was no effect on action potential-independent excitatory postsynaptic event rates and excitatory synaptic strength between DIV 10 and 15.

## Discussion

**Figure 5:**
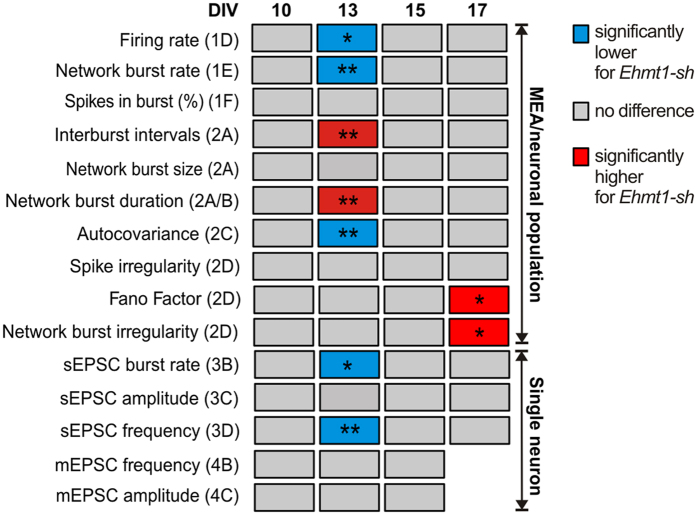
Fingerprint of the functional maturation of EHMT1-deficient vs. control networks. Summary of the statistics derived from electrophysiological recordings of action potential activity at the network level (MEA), action potential-dependent excitatory input at the single cell level (sEPSC) and excitatory basal synaptic transmission at the single cell level (mEPSC). Together these data give a fingerprint of the developmental effect of EHMT1 deficiency. In general, EHMT1-deficient neuronal cultures showed reduced action potential activity at DIV 13, which at DIV 15 and 17 recovered to match the statistics of the control networks. At DIV 17 the EHMT1-deficient networks have increased values for measures representing the irregularity in the timing of the bursts. P-values were corrected for multiple comparisons using the false discovery rate method (see Supplemental Table S1).

In this study we used extracellular and intracellular recordings in developing cortical networks to investigate the time course of the effects of loss of EHMT1 (Fig. 5). We demonstrated that the emergence of spontaneous network activity was delayed in cortical neurons deficient in EHMT1 compared to control conditions. This phenotype was also observed at the single neuron level in terms of a transient reduction in the frequency of spontaneous excitatory input during development, which recovered by the end of the recording period. Finally, we showed that the transient delay in spontaneous network activity in EHMT1-deficient networks was followed by an increased network burst irregularity later in development, whereas our control networks showed periodic and rhythmic bursting, which is typical for cultured neurons^28^,^41^.

A network burst emerges stochastically: cells are spontaneously, but independently and irregularly active and the generation of an action potential depends on whether synaptic inputs and noise depolarize the cell sufficiently to exceed the voltage threshold^43^. In *in vivo* networks, other mechanisms exist to adjust network excitability such as the neuromodulatory tone or oscillations used to direct communication^43^,^44^. When enough cells fire in coincidence, excitation to the other cells can generate a run-away process (burst) in which most cells are activated, often multiple times. Pacemaker cells are intrinsically active, regularly firing neurons^45^ that were shown to best simulate the generation of network bursts in neuronal cultures^46^. Synaptic dynamics are furthermore important for burst behaviour, where bursts terminate when the synaptic vesicle pool is depleted^46^,^47^. This determines the duration of the burst and how long the recovery takes. We found an increased irregularity in the timing at which network bursts occurred for the EHMT1-deficient networks relative to control networks at DIV 17. The irregularity of network bursts is thought to be related mainly to two factors. First, to the stochastic mechanism by which bursts are initiated, i.e. differences of coincidences in EHMT1-deficient networks or regulatory pacemakers. Second, irregularity depends on the topological pattern of neuronal wiring, i.e. less efficient propagation through the network and less effective recruitment of other neurons^38^,^39^,^46^. At DIV 17 we found a comparable firing rate, relevant for coincidences, and burst rate between conditions. Furthermore, the regularity of the spike timing was not different between conditions as indicated by the coefficient of variation of the ISIs, suggesting that in our data there was no major contribution of pacemaker cells to the generation of the more irregular bursting patterns. Together, our results suggest that the increased irregularity in EHMT1-deficient networks can be related to differences in network topology that may originate from altered network formation during the early development. However, further investigation using high-density electrode devices and spike sorting methodology will be required to clarify whether and to which extent pacemaker cells may contribute to the irregular pattern of activity in EHMT1-deficient networks.

At the early developmental stage we observed a reduced network burst rate and prolonged network burst duration; this could be related to a reduction in action potential-dependent vesicle release in the EHMT1-deficient cultures during early development. We previously showed a decrease in release probability at the CA3-CA1 synapse in *Ehmt1*^+/−^ mice^19^. Likewise, in our current study in cortical neurons we found a decrease in action potential-dependent input, which could thus underlie the deficits in spontaneous network activity. These results clearly indicate that EHMT1-deficiency relates to an impaired action potential-dependent vesicle release, which is a calcium-dependent process. Here, we did not observe differences between conditions in mEPSCs, which is the calcium-independent process of vesicle release. The two modes of calcium-(in)dependent vesicle release involve different sets proteins in the axonal boutons (for review, see ref. 48). To further investigate the differential regulation by EHMT1 of these modes, it will be of interest to study expression patterns of the involved proteins.

The impaired action potential-dependent synaptic transmission occurred during the transition from uncorrelated spiking to network-wide burst spiking, which coincided with the time point at which hippocampal neuronal cultures switch from the mode of release from exclusively spontaneous early on in development to predominantly evoked in mature neurons^40^. We previously showed that this transition can initiate a period of rapid rewiring in brain circuits^49^. Impairments in action potential activity and synaptic transmission could thus result in miswiring of the circuitry^24^.

Indeed, neuronal activity plays an important role in refinement of synaptic connections (for reviews see refs 50 and 51). Activity-dependent forms of synaptic plasticity, in particular, Hebbian and homeostatic forms of plasticity, guide the cortical refinement and are required for functional maturation of cortical circuits^52^. Recent data suggest the existence of developmental phenotypic checkpoints that, if not met, prevent further development. Misregulation during these time-windows causes cellular and network disturbances that underlie several phenotypes associated with neurodevelopmental disorders, including ID and autism^25^,^53^,^54^ and give rise to behavioral symptoms^24^. Given that sequentially activated gene expression programs underlie the genomic programs of synapse function^35^, epigenetic regulation of gene transcription is highly suited to control genetic programs and gene expression during development. It is therefore of interest to note that EHMT1 has recently been found to be important for activity-dependent remodeling of synapses and circuitry during learning and memory as well as in the context of addiction^11^,^20^,^55^. We recently found that EHMT1 deficiency, in a cell-autonomous way, impairs homeostatic synaptic scaling in cortical neurons *in vitro* and *in vivo*^23^. Homeostatic synaptic scaling is a form of plasticity that is used by neurons to adjust synaptic strength during development in order to maintain neuronal activity within a certain range (Turrigiano, 2008). To reach this level of activity, which we refer to as the ‘target activity level’, cells use sensors to detect the level of spiking activity; if the measured activity level deviates from the target activity level, compensatory mechanisms are activated accordingly^56^. Here we show that neuronal network activity was lower during early development for EHMT1 deficient neurons, but synaptic strength was not adjusted accordingly. We therefore hypothesize that in EHMT1-deficient neurons network abnormalities can primarily be attributed to an impairment in homeostatic synaptic scaling and/or detection of cellular activity. Deficiencies at the network level have also been observed in other neurodevelopmental disorders, including in Tuberous Sclerosis Complex (TSC), which is caused by loss-of-function mutations in the mTOR negative regulators *TSC1* or *TSC2*. In *Tsc1* KO neuronal cultures, network hyperexcitability was attributed to a primary imbalance in excitation and inhibition due to reduced inhibition onto pyramidal neurons, but not to alterations in homeostatic excitatory synaptic plasticity^33^. A better understanding of which perturbations are directly causal and which are induced secondarily as a consequence of altered brain function will be essential to further our mechanistic understanding of neurodevelopmental disorders.

In conclusion, we showed that deficiency of the epigenetic factor EHMT1 impaired spontaneous electrophysiological activity during the transition from uncorrelated background spiking activity to synchronized network bursting. During this developmental stage both synchronized network bursting as well as action potential-dependent excitatory input to the neurons was reduced. The impairment in neuronal activity early in development could relate to inappropriate wiring and irregular behavior later in development and thereby contribute to the pathophysiology of Kleefstra syndrome.

## Material and Methods

### DNA constructs and virus production

For RNAi knockdown experiments, DNA fragments encoding short hairpin RNAs (shRNAs) directed against mouse/rat *Ehmt1* mRNA (*Ehmt1-sh*: 5′-GGTGATTGAGATGTTTAA-3′; and control shRNA (*control-sh*: 5′-GCTCACCCTTCCTACTCTC-3′)^23^ were cloned into the pSuper vector (Oligoengine) and pTRIPΔU3-EF1α-EGFP lentiviral vector^57^,^58^. Since no differences were observed between *control-sh* and uninfected cells, data from both conditions were pooled and noted as ‘control’ throughout the manuscript. Lentiviral supernatants were prepared, concentrated, and titered as described^57^,^59^. Briefly, lentiviruses were generated by co-transfecting the transfer vector, the HIV-1 packaging vector Δ8.9, and the VSVG envelope glycoprotein vector into HEK293T cells, using calcium phosphate precipitation. Supernatants of culture media were collected 48-h after transfection and centrifuged at 100,000 × g to concentrate the viral vector. Viral particles were then stored at −80 °C until use.

### Cell culture

All experiments on animals were carried out in accordance with the approved animal care and use guidelines of the Animal Care Committee, Radboud University Nijmegen Medical Centre, the Netherlands, (RU-DEC-2011-021, protocol number: 77073). The day before plating of cells, micro-electrode arrays (MEAs) and glass cover slips (14 mm, Menzel GmbH) treated with 65% nitric acid (Sigma-aldrich, 84380) were coated with 0.0125% Polyethylenimine (PEI, Sigma Aldrich P3143) overnight. After a triple wash to remove residual PEI, a seeding medium containing Neurobasal medium (1x, Gibco, 21103–049), 10% Fetal Bovine Serum (FBS; Sigma, F7524), 2% Supplement B27 (B27, Gibco, 17504–044) and 1% Penicillin/Streptomycin (Pen/Strep; Sigma, P4333) was added to the MEAs and cover slips. Cortices were dissected from Wistar rat pups on embryonic day 18 (E18) as described^60^. The cells were plated with a cell density of 60,000 cells/cm^2^ (low density culture) for MEA recordings and of 50,000/cm^2^ for whole-cell patch-clamp recordings. 4 hours after plating half of the medium was replaced by culturing medium containing Neurobasal Medium with 2% B27, 1% Pen/Strep and 1% GlutaMAX (Gibco, 35050-038). At 24 hours after plating, the cells were infected with a virus expressing GFP and an shRNA (*Ehmt1-sh* or *control-sh*). To determine the titer of each virus batch, neurons were infected at DIV 1 and imaged for GFP at DIV 7. In parallel cells were prepared for western blot analysis to measure the efficiency of knockdown (Supplemental Fig. S1) on DIV 7. To assess the level of apoptosis, neuronal cultures were incubated with propidium iodide (Sigma Aldrich) at DIV 7. None of the virus used (control and *Ehmt1-sh)* induced apoptosis (data not shown).

Cortical cultures from mice were prepared from individual E16.5 embryos. Since the genotype was unknown at the time of harvest, each embryo was collected and the brains were processed separately. Each whole brain was kept on ice in 1 mL L-15 medium, organized separately in a 24-well plate, and tail clips were collected for genotyping. The cortical hemispheres isolation, dissociation and cell plating was performed as described for the rat.

### Micro-electrode array recording

The spontaneous activity was recorded at DIV 10, 13, 15 and 17 for 20 minutes by means of MEAs. MEAs consisted of 60 titanium nitride planar electrodes (model 4QMEA1000, Multi Channel Systems, Reutlingen, Germany) that were embedded in a glass substrate and insulated by silicon nitride. Recordings were performed using the USB-MEA60-Inv-BC-System (Multi Channel Systems, Reutlingen, Germany). Signals were recorded at a sampling frequency of 10 kHz. During recordings cultures were kept in humidified atmosphere of 95% O_2_ and 5% CO_2_ at 37 °C (temperature controller TC02, Multi Channel Systems, Reutlingen, Germany). The rat cortical neuron data were obtained from 9 different batches of cells, while the mouse data represents the accumulation of 6 litters. All recordings and analysis were performed blind for genotype.

### Whole-cell patch-clamp recordings

The cover slips containing the primary cortical cultures were transferred to a submerged fixed-stage recording chamber in an upright microscope (BX51WI, Olympus, Hamburg, Germany). Recordings were performed under submerged conditions in oxygenated (95% O_2_ and 5% CO_2_) Artificial Cerebrospinal Fluid (ACSF) containing (in mM): 124 NaCl, 1.25 NaH_2_PO_4_, 3 KCl, 26 NaHCO_2_, 11 Glucose, 2 CaCl_2_, 1 MgCl_2_, adjusted to pH of 7.4 with NaOH, at 30 °C. Neurons were recorded using borosilicate glass patch pipettes (electrode resistance 5–6 MΩ) filled with a potassium-based solution for current clamp experiments containing (in mM) 130 K-Gluconate, 5 KCl, 10 HEPES, 2.5 MgCl_2_, 4 Na_2_-ATP, 0.4 Na_3_-ATP, 10 Na-phosphocreatine, 0.6 EGTA and a cesium-based solution for voltage clamp experiments containing (in mM) 115 CsMeSo3, 20 CsCl, 10 HEPES, 2.5 MgCl2, 4 Na2ATP, 0.4 NaGTP, 10 Na-Posphocreatine, 0.6 EGTA both adjusted to a pH of 7.4 and to approx. 304 mOsmol. Electrophysiological data were not corrected for a junction potential of ca. −10 mV.

Intrinsic electrophysiological properties were recorded in current clamp mode. Only cells showing a resting membrane potential <−50 mV and stable passive and active properties during recording were taken into consideration. Passive intrinsic membrane properties were determined via injection of a 0.5 s hyperpolarizing current of −25 pA at a holding potential of −60 mV. At the same holding potential action potential (AP) characteristics were determined from the first AP elicited by a 0.5 s depolarising current just sufficient to bring the membrane potential of the cell to threshold. The rheobase was determined by the amount of current required to generate this first AP. AP threshold was defined as the voltage at which the slope of the membrane potential strongly increases and exceeds 10 mV/ms.

Miniature excitatory postsynaptic currents (mEPSC) were measured in voltage clamp at a holding potential of −60 mV in the presence of 1 μM tetrodotoxin (Tocris, Bristol, UK) and 100 μM picrotoxin (Tocris, Bristol, UK). Spontaneous action potential-evoked postsynaptic currents (sEPSC) were recorded in ACSF without additional drugs at a holding potential of −60 mV. Synaptic event detection was performed with MiniAnalysis (Synaptosoft Inc, Decatur, GA, USA). For the traces that contained strongly accumulated sEPSCs mediated by presynaptic synchronized bursts of action potentials, these bursts of sEPSCs were counted by visual inspection.

### MEA data analysis

Data were processed and analyzed using an automated, scripted procedure developed in MATLAB (The Mathworks, Natick, MA, USA).

#### Spike detection

After data high-pass filtering (300 Hz, 6^th^ order Butterworth) and offset correction (the median of the absolute value of the signal was subtracted for each recording to center the signal around zero volt), spike detection was performed as previously described in ref. 27, using a peak-to-peak detection threshold (Θ_ap_)^61^, calculated as: Θ*ap* = *f *∙ M (|*signal*|) ∙ 1.5.

with f being the gain factor (f = 8) and M(|signal|) the median of the absolute value of the signal.

To prevent variability due to differences in the number of active channels during development, the *mean firing rate* (MFR) of every network at each time point was obtained by counting the number of spike detected from each channel and dividing the values by the number of active electrodes at DIV 17 (i.e, being an active electrode defined by an activity >0.1 spike/s).

#### Separating bursts and background action potentials

Only active electrodes (i.e. firing rate > 0.1 Hz) were taken into account. Channels with noise and very high firing rate were discarded (see Supplemental Fig. S3). Network bursts and background action potentials were separated using a 2-step procedure^27^: 1) action potentials for the individual MEA electrodes were labeled as background action potentials if the pre- and post ISI exceeded a threshold (θ_1_ = 100 ms); 2) action potentials from all electrodes were combined into a single action potential train. If the pre- and post ISIs exceeded a second threshold (θ_2_ = 5 ms), action potentials were also labeled as background action potentials. A network burst was detected when the spike density trace crossed a predefined threshold of 10 Hz from below for 60 active channels. The action potentials were convolved with a Gaussian function:


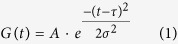


where τ is the time of the spike, A is the amplitude, which was set to 1 and σ is the width of the Gaussian, which was set to 50 ms.

#### Burst duration, size and interburst interval (IBI)

The burst duration is taken as the time between spike density crossing 10 Hz from below up to when the activity again drops below 10 Hz. The burst size is defined as the area under the spike density trace for the burst duration, divided by the surface area of the Gaussian with which each spike was convolved (Equation (1)). The IBI is the time between two consecutive bursts. Cumulative probabilities for the burst size, IBI and burst duration were obtained using logarithmically spaced bin sizes (normalization factor is shown in Fig. S4A).

#### Spike time irregularity

We tested two measures of spike irregularity: (1) the coefficient of variation (CV) of the interspike intervals (ISI), which is the standard deviation divided by the mean of the ISI. The CV was based on active electrodes only. And (2), the Fano Factor (FF), a measure of spike count dispersion:


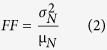


With σ_N_ being the standard deviation and μ_N_ being the mean of the number of action potentials on an active electrode averaged across different segments, each 5 seconds of duration. A sliding window applied at intervals of one second was used to calculate the number of action potentials for each segment. The CV_ISI_ and FF were averaged over the active electrodes and calculated separately for each DIV and culture.

*Burst irregularity.* We tested two measures of burst irregularity, (1) the coefficient of variation (CV) of the IBIs as defined above and (2), a burst rate independent measure for irregularity (IR):


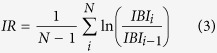


With *i* being the index of the IBI and N the number of IBIs^42^. The CV_IBI_ and IR measures were calculated separately for each DIV and culture.

#### Autocovariance

The autocovariance was calculated as:


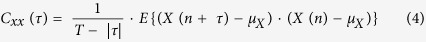


Where C_xx_(τ) is the autocovariance for action potentials on electrode *x* at time lag *τ* (where 0 < |*τ*| < 2 seconds), where the expectation E is taken across samples *n* with *μ*_*x*_ being the mean firing rate of that electrode. The autocovariance was normalized by the length of the recording (*T*), which was adjusted by the absolute time lag |*τ*|. Due to the discretization at a high sampling rate (10 kHz), the autocovariance was smoothed across *τ* with a 5 ms moving average. To perform statistics, we calculated the autocovariance half-width, which is the width at which the autocovariance is equal to half its maximal value. The peak of the autocovariance was normalized for each recording separately (normalization factors in Fig. S4B).

### Statistical tests

Data are expressed as mean ± standard error of the mean. Statistical analysis was performed using MATLAB (R2012a, The Mathworks, Natick, MA, USA). The data submitted to statistical analysis was often (59%) non-normal, as indicated by skewness (we tested for normality using the Jarque-Bera test, implemented as jbtest in MATLAB). Hence, we used the Mann–Whitney test, implemented as ranksum in MATLAB. We tested whether the means of the set of statistics calculated from the MEA and whole-cell patch-clamp recordings differed across conditions for any given DIV. These p-values were aggregated for each DIV value; significant values were then corrected for multiple comparisons using the false discovery rate (FDR) method, incorporating potential dependencies between p-values^34^,^62^. To calculate the FDR we used the mafdr function in MATLAB using the polynomial selection method for the parameter lambda. Significance levels after correcting for multiple comparisons were set at p < 0.05. FDR was set to not increase significance level, which could occur at DIV 13 due to the strong significance found in many parameters.

## Additional Information

**How to cite this article**: Martens, M. B. *et al*. Euchromatin histone methyltransferase 1 regulates cortical neuronal network development. *Sci. Rep.* **6**, 35756; doi: 10.1038/srep35756 (2016).

## Supplementary Material

Supplementary Information
