# Supplementary material for: Euchromatin histone methyltransferase 1 regulates cortical neuronal network development
Source: Sci Rep. 2016 Oct 21;6:35756. doi: 10.1038/srep35756 (PMC5073331; doi:10.1038/srep35756)
Supplement: Supplementary Information [file srep35756-s1.pdf]

Euchromatin histone methyltransferase-1 regulates cortical neuronal network development

Marijn Martens[1,4,5], Monica Frega[2,4], Jessica Classen[2,4], Lisa Epping[1], Elske Bijvank[2,4], Marco Benevento[2,4], Hans van Bokhoven[2,3,4], Paul Tiesinga[1,4,*], Dirk Schubert[2,4,*] and Nael Nadif Kasri[2,3,4,5 *]

[1]Department of Neuroinformatics, Radboud University Nijmegen
[2]Department of Cognitive Neuroscience, Radboudumc, P.O. Box 9101, 6500 HB, Nijmegen, the Netherlands
[3]Department of Human Genetics, Radboudumc, P.O. Box 9101, 6500 HB, Nijmegen, the Netherlands
[4]Donders Institute for Brain, Cognition and Behaviour, P.O. Box 9101, 6500 HB, Nijmegen, the Netherlands
[5]corresponding authors
*co-senior authorship

Supplemental Material

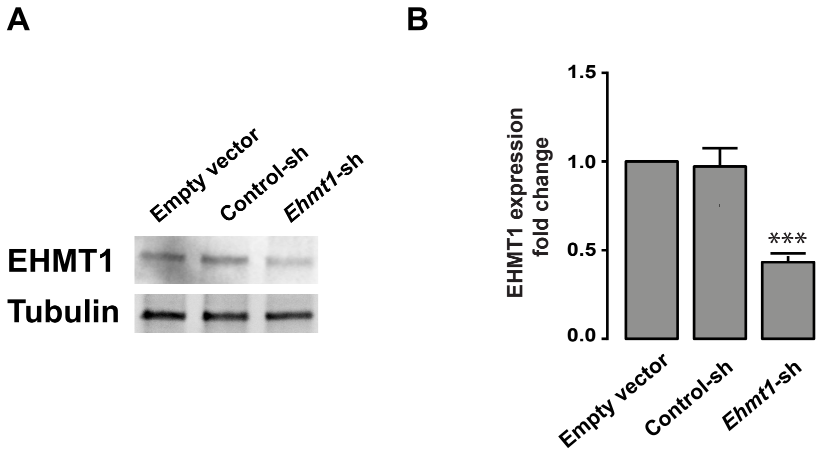

Supplemental Figure S1. Validation of *Ehmt1* knockdown. A: Primary cortical neurons were transduced with empty vector, control-shRNA or *Ehmt1*-sh expressing lentivirus at DIV1. Protein extracts were prepared at DIV7 and were probed by immunoblotting with antibodies against EHMT1 and Tubulin. **B:** Quantification of the immunoblot signals of (A). Data are represented as mean ± SEM, n=3; ***, p < 0.001; t-test.

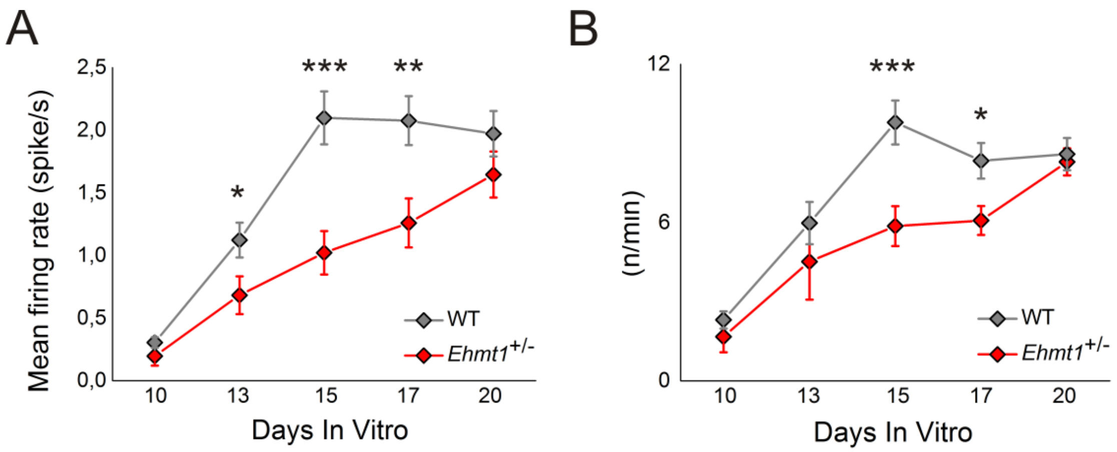

Supplemental Figure S2. Electrophysiological activity of cultures on MEAs from wild-type (WT) and *Ehmt1*$^{+/-}$ mice during development. Mean firing rate (**A**) and burst rate (**B**) during development for both genotypes.

Data are means ± SEM. Statistics were based on n=12 recordings for control and n=10 for *Ehmt1*$^{+/}$.
* denotes $p < 0.05$, ** denotes $p < 0.01$, *** denotes $p < 0.001$ (Mann–Whitney test).

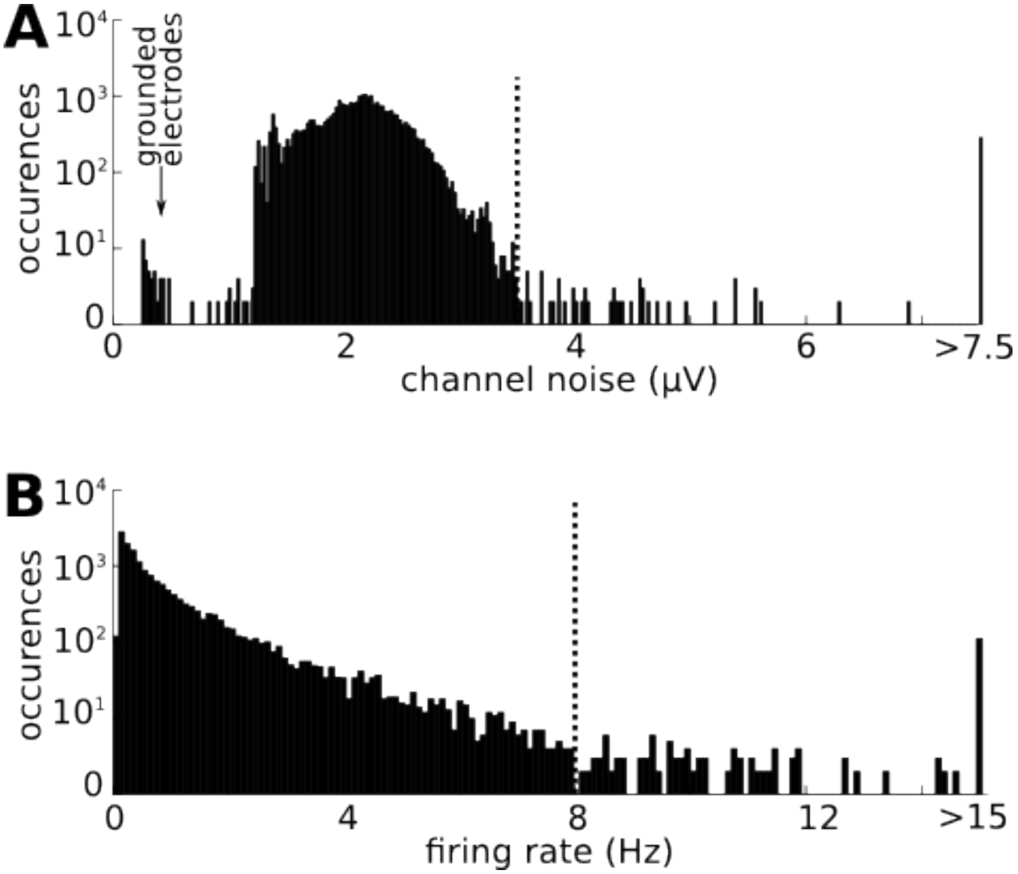

Supplemental Figure S3. **Channel noise and firing rate. A**: Semi-logarithmic plot of channel noise on the MEA electrodes. High noise can be caused by damage or bad contact to the recording device. Electrodes were discarded when the absolute of the median of the noise exceeded 3.5 µV (denoted by dotted line), which happened for 1.3% of recorded electrodes. The number of channels with noise that was discarded was not significantly different between conditions. **B**: Semi-logarithmic plot of the firing rates. Occasionally very high firing rates are detected, potentially from artifacts. Firing rates that exceeded 8 Hz (denoted by dotted line) were discarded, which occurred on 0.7% of the recorded electrodes. The number of channels with high firing rates that were discarded was not significantly different between conditions.

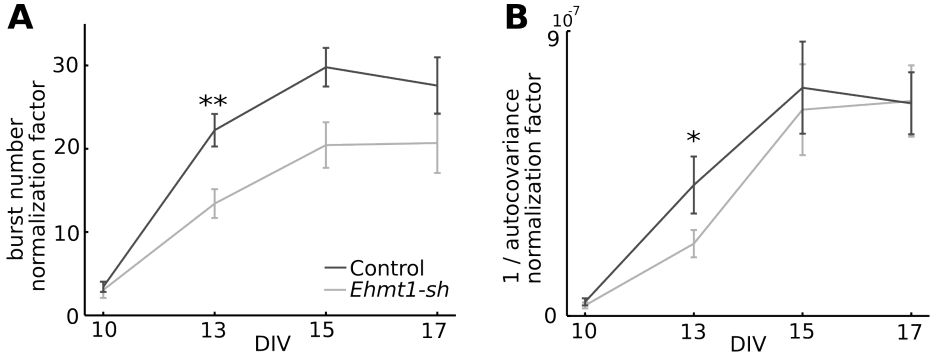

Supplemental Figure S4. **Normalization factors. A**: The cumulative probabilities (Figure 2A) were normalized such that the relative shape of the distribution can be compared. **B**: Normalization factors for the peak of the autocovariance distributions. Data are means ± SEM. Statistics were based on n = 24 (DIV 10), 25 (DIV 13), 25 (DIV 15) and 24 (DIV 17) recordings for control and n = 19 (DIV 10), 19 (DIV 13), 19 (DIV 15) and 18 (DIV 17) for *Ehmt1*-sh DIV; * denotes $p < 0.05$, ** denotes $p < 0.01$ (Mann–Whitney test, with p-values corrected for multiple comparisons using the false discovery rate method).

		After multiple test correction			
**DIV**		**10**	**13**	**15**	**17**
Firing rate (1D)		0.7824	**0.0371**	1.000	0.8092
Network burst rate (1E)		0.5182	**0.0031**	0.1472	0.1660
Spikes in burst (%) (1F)		0.5248	0.1221	0.5632	0.6162
Interburst intervals (2A, first panel)		0.9886	**0.0048**	0.2848	0.1584
Network burst size (2A, second panel)		0.4520	0.5696	0.7555	0.2177
Network burst duration (2A, third panel)		1.000	**0.0006**	0.1864	0.1071
Firing during network burst (2B)		1.000	**0.0006**	0.1864	0.1071
Autocovariance (2C)		0.7527	**0.0007**	0.6296	0.3093
Spike irregularity (2D, first panel)		0.6308	0.3807	0.5171	0.1241
Fano Factor (2D, second panel)		0.6819	0.0612	0.9811	**0.0402**
Network burst irregularity (2D, third panel)		1.0000	0.3555	1.0000	**0.0495**
sEPSC burst rate (3B)		0.4067	**0.0219**	0.1909	0.9767
sEPSC amplitude (3C)		0.7323	0.5200	0.5401	0.2801
sEPSC frequency (3D)		1.0000	**0.0043**	0.6357	0.4655
mEPSC amplitude (4B)		0.5680	0.9461	1.0000	
mEPSC frequency (4C)		0.4837	0.3040	1.0000	

Supplemental Table S1: P-values for the MEA and single neuron recordings, using the Mann–Whitney test, implemented as ranksum in MATLAB. Significant values were corrected for multiple comparisons using the false discovery rate (FDR) method, incorporating potential dependencies between p-values. To calculate the FDR we used the mafdr function in MATLAB using the polynomial selection method for the parameter lambda. Significance levels after correcting for multiple comparisons were set at $p < 0.05$, here shown in the bold font. FDR was set to not increase significance level, which could occur at DIV 13 due to the strong significance found in many parameters.

**Electrophysiological parameter**	**Control neurons**	**EHMT1-deficient neurons**
	(N = 6)	(N = 7)
Resting membrane potential (mV)	-54.7 ± 1.4	-56.9 ± 1.8
Capacitance (pF)	102.1 ± 6.2	94.2 ± 5.0
Input resistance (MΩ)	296.3 ± 25.5	**403.7 ± 37.2***
Time constant (ms)	30.3 ± 3.3	38.0 ± 3.5
Rheobase (pA)	70.0 ± 11.2	**46.4 ± 9.0***
Threshold (mV)	-32.6 ± 2.2	-30.8 ± 1.3
Action potential amplitude (mV)	61.3 ± 3.7	61.3 ± 2.6
Action potential half-width (ms)	2.5 ± 0.4	2.1 ± 0.3

Supplemental Table S2
Intrinsic electrophysiological properties of control and EHMT1-deficient neurons at DIV 13
Values represent mean ± SEM. DIV: days in vitro; Asterisks (*) show significant difference between control neurons and Ehmt1-deficient neurons with * = $p < 0.05$. Electrophysiological parameters were determined as described in the materials and methods section.
